# Self-compassion in migraine: associations with depression, anxiety, and clinical characteristics

**DOI:** 10.1055/s-0046-1820105

**Published:** 2026-04-28

**Authors:** Ruken Simsekoglu, Zarife Tugce Yıldız, Nilay Garipbas

**Affiliations:** 1 Istanbul Göztepe Prof. Dr. Suleyman Yalcın City Hospital, Department of Neurology, Istanbul, Turkey.; 2 Istanbul Göztepe Prof. Dr. Suleyman Yalcın City Hospital, Department of Psychology, Istanbul, Turkey.

**Keywords:** Migraine Disorders, Pain, Self-Compassion, Mindfulness, Depression, Anxiety

## Abstract

**Background:**

Self-compassion (SC), defined as a positive and accepting attitude towards oneself during times of suffering or failure, is receiving more attention in the context of chronic pain disorders. However, very little is known regarding the link between SC, psychological symptoms, and migraine features.

**Objective:**

To compare the SC levels of migraine patients to those of healthy controls and investigate the relationships among SC, anxiety, depression, and migraine features.

**Methods:**

This cross-sectional study comprised 90 healthy controls (age: 35 ± 9.8 years, 75 female patients) and 98 migraine sufferers (age: 36.7 ± 11.3 years, 84 female patients). The Beck depression inventory, Beck anxiety inventory, and self-compassion scale were completed by the participants.

**Results:**

The SC levels were considerably lower in migraine patients. Patients with chronic migraine performed worse than those with episodic migraines on the self-kindness, common humanity, and mindfulness subscales. Patients with medication overuse also had reduced SC. Higher isolation scores were linked to aura presence.

**Conclusion:**

We found impaired SC in individuals with migraine, and a connection with greater emotional distress and migraine characteristics. These results demonstrate the possible value of emphasizing SC in migraine therapies, especially for those who suffer from chronic migraine, aura, or medication overuse.

## INTRODUCTION

Migraine is a chronic paroxysmal neurological disorder represented by moderate to severe headache episodes, frequently leading to significant disability and reduced quality of life. 1 Episodic migraine is characterized by recurrent headaches usually accompanied with nausea, vomiting, photophobia, and phonophobia, whereas chronic migraine is defined as headaches occurring on 15 or more days per month, with a minimum of 8 days exhibiting classic migraine symptoms. 1 2

Around 14% of people worldwide struggle with migraines, which are a serious public health concern with significant socioeconomic consequences. 2 3 It is one of the most prevalent neurological conditions, with a lifetime frequency of up to 33% in women and 13% in males. 2 3 4 Even though migraine is highly prevalent, the underlying etiology is still complex and poorly understood, combining genetic, physiological, environmental, and psychological factors. 5 6 7 8

Psychological stress and negative emotional states such as distress, sadness, and anxiety are known migraine triggers, and may intensify the severity or duration of attacks. 9 Furthermore, people with migraines are more likely than the general population to suffer from anxiety and depression. 10 Given the bidirectional relationship between migraine and mood disorders, cognitive-behavioral and mindfulness-based therapies have been effectively used as adjuncts to pharmacological treatment. 10 11 Self-compassion (SC) has recently been a relevant therapeutic target for migraine and other chronic pain conditions. 11 12

Throughout difficult circumstances, SC requires treating oneself with the same thoughtfulness and warmth that one would show a good friend, rather than with judgement or coldness. 13 It also requires maintaining a healthy emotional awareness and accepting that individual hardships are a natural part of the human experience. 13 14 Therefore, SC is characterized by a conscious, nonjudgmental disposition towards oneself in moments of failure or distress, incorporating self-kindness, recognition of shared human experience, and emotional balance. 13 14 15 16 Elevated levels of SC correlate with enhanced psychological well-being and diminished symptoms of despair and anxiety. 17 18 In persons with chronic pain, increased SC is associated with less pain catastrophizing and emotional distress. 19

Nevertheless, there is an absence of studies on SC in migraineurs. A better understanding of the emotional and cognitive features of this group may be possible by investigating the relationship between SC and migraine diagnoses, also associated psychological symptoms and migraine traits (such as chronicity, aura, and medication overuse).

The primary aim of this study was to assess levels of SC in patients with migraine compared to healthy controls. Additionally, we aimed to explore the associations between SC, depression and anxiety symptoms, and migraine traits. We hypothesized that migraine patients would report lower SC compared to healthy controls; these lower results would be associated with higher levels of depression and anxiety; and SC would vary depending on migraine chronicity, presence of aura, and medication overuse.

## METHODS

This cross-sectional study included 98 adult patients diagnosed with migraine who presented to the Headache Clinic of the Neurology Department at the Istanbul Göztepe Prof. Dr. Süleyman Yalçin City Hospital, between November 2023 and March 2024. Migraine diagnoses were established according to the 2018 International Classification of Headache Disorders, 3rd edition (ICHD-3), 1 including those with and without aura. Patients were further classified as having episodic or chronic migraine based on headache frequency (< 15 or ≥ 15 headache days/month, respectively). Although “episodic migraine” is not a formal diagnostic category in ICHD-3, this terminology was used for descriptive purposes to differentiate between subgroups.

A control group of 90 healthy individuals was recruited during the same study period from the hospital staff and relatives of the hospital staff and research team. This group was frequency-matched to the patient group in terms of age, gender, and educational level to enhance comparability and reduce potential selection bias. All controls underwent a brief clinical interview conducted by the same neurologist and a review of medical history to confirm the absence of any current or past neurological or psychiatric disorders, as well as any chronic pain condition. Individuals with a history of migraine, or other recurrent headache disorders, chronic pain conditions, or current psychotropic medication use, were excluded.

Clinical interviews and diagnostic evaluations were conducted by the same neurologist (blinded to questionnaire scores), who also collected disease-specific data.

Medication overuse was defined as the regular intake of non-steroidal anti-inflammatory drugs on ≥ 15 days per month or triptans on ≥ 10 days per month for at least 3 months, in accordance with ICHD-3 criteria. Medication overuse headache (MOH) was defined as a headache occurring on ≥15 days per month that developed as a consequence of medication overuse in a patient with a preexisting primary headache disorder.

Patients fulfilling diagnostic criteria for MOH were included in the study and analyzed as a separate subgroup. The severity of migraine attacks was self-rated using a 10-point visual analog scale (VAS).

Inclusion criteria were a confirmed migraine diagnosis according to ICHD-3 and voluntary participation. On the other hand, the exclusion criteria were presence of any other neurological disorder; history or current diagnosis of a major psychiatric disorder based on clinical interview and medical records; coexisting primary headache disorders other than infrequent episodic tension-type headache; having an acute migraine attack during data collection; and pregnancy or breastfeeding.

All participants completed the sociodemographic data form, the Beck depression inventory (BDI), the Beck anxiety inventory (BAI), and the self-compassion scale (SCS) under the supervision of a trained clinical psychologist in a quiet laboratory setting.

The study was approved by the Local Ethics Committee of the hospital. The register number of the ethics committee was 2023/0759. All procedures were conducted in accordance with the Declaration of Helsinki and its later amendments. Written informed consent was obtained from all participants.

### Assessment tools

A sociodemographic and clinical data form was used to collect data included participants' age, gender, education, migraine duration (in years), presence of aura, presence of chronic migraine, medication overuse, and family history of headache.

The BDI is a 21-item self-report inventory assessing depressive symptoms in adults. 20 Items are scored from 0 to 3, with higher scores reflecting more severe depression. The Turkish adaptation was validated by Hisli; a cutoff score of 17 was used. 21

The BAI is a 21-item scale measuring the frequency of anxiety symptoms. 22 Each item is rated 0 to 3; higher scores indicate greater anxiety. The Turkish version has demonstrated good validity and reliability. 23

The self-compassion scale (SCS) was developed by Neff, 24 this 26-item scale evaluates six components of SC: self-kindness, self-judgment, common humanity, isolation, mindfulness, and over-identification. Items were rated on a 5-point Likert scale. 24 The total SC score was calculated by reverse-scoring the negative subscales (self-judgment, isolation, and over-identification) and then summing all 26 items, with higher scores indicating greater SC (range: 26–130). The Turkish version was validated by Akın et al., with subscale reliabilities ranging from 0.72 to 0.80. 25

### Statistical analysis

All statistical analyses were conducted using the IBM SPSS Statistics (IBM Corp.), version 27.0. Descriptive statistics included means, standard deviations (SDs), medians, ranges, frequencies, and percentages. Normality of data distribution was assessed using the Shapiro-Wilk test separately for each group and for each continuous variable. Depending on the distribution, comparisons between groups were made using either the independent samples t-test for normally distributed variables, or the Mann–Whitney U test for non-normally distributed variables. Chi-square tests were used for categorical variables. In the migraine group, Spearman's rank correlation was performed to examine the relationships between SC subscale scores and clinical variables (e.g., duration of migraine, depression, and anxiety scores).

Hierarchical linear regression analysis was conducted to examine whether migraine status was independently associated with SC after controlling for demographic variables and psychological symptoms. The subscale's total score was entered as the dependent variable.

In Step 1, age, gender (coded as 1 = female, 2 = male), and years of education were entered as demographic covariates. In Step 2, the results from BAI and BDI were added to the model to account for psychological distress. In Step 3, migraine status (migraine vs. healthy control) was entered to examine its incremental contribution beyond demographics and psychological symptoms. Multicollinearity was evaluated using tolerance and variance inflation factor (VIF) values. All tolerance values exceeded .80 and all VIF values were below 1.20, indicating no multicollinearity concerns. Statistical significance was set at *p* < 0.05 (two-tailed).

## RESULTS

### Characteristics of migraine patients

Among the 188 participants included in the study, 98 were migraine patients (mean age: 36.7 ± 11.3 years; 84 female patients) and 90 were healthy controls (mean age: 35.7 ± 9.8 years; 75 female patients). The mean duration of migraine was 11.4 ± 9.0 years. Patients rated the average intensity of migraine attacks as 7.8 ± 0.9 on the Visual Analog Scale (VAS). A positive family history of migraine was reported by 88.8% (n = 87) of patients. Episodic migraine was observed in 90.8% (n = 89), and 9.2% (n = 9) met the criteria for chronic migraine according to the ICHD-3. Migraine with aura was present in 21.4% (n = 21), including both patients with exclusively aural attacks and those experiencing both aural and non-aural episodes. Medication overuse, in line with the established criteria, was identified in 10.2% (n = 10) of patients. Detailed clinical characteristics are summarized in Table 1 .

**Table 1:** Migraine characteristics of patients

		Minimum–maximum			Median	Mean ± SD / n (%)		
Migraine time		1.0–45.0			8.0	11.4 ± 9.0		
VAS score		6.0–10.0			8.0	7.8 ± 0.9		
Medication overuse	(-)					88 (89.8%)		
	(+)					10 (10.2%)		
Family history of migraine	(-)					11 (11.2%)		
	(+)					87 (88.8%)		
Migraine type	Episodic					89 (90.8%)		
	Chronic					9 (9.2%)		
Migraine aura	(-)					77 (78.6%)		
	(+)					21 (21.4%)		

Abbreviations: SD, standard deviation; VAS, visual analog scale.

### Comparison of clinical scale scores between migraine patients and healthy controls

**Figure 1:**
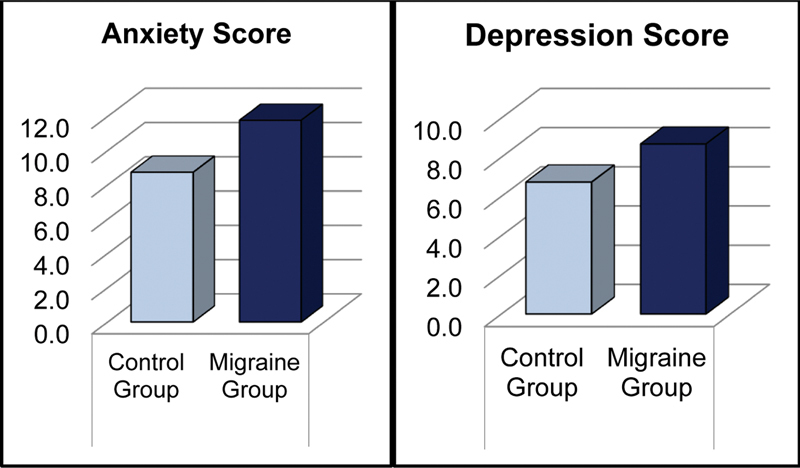
Anxiety and depression levels in migraine and healthy controls.

**Figure 2:**
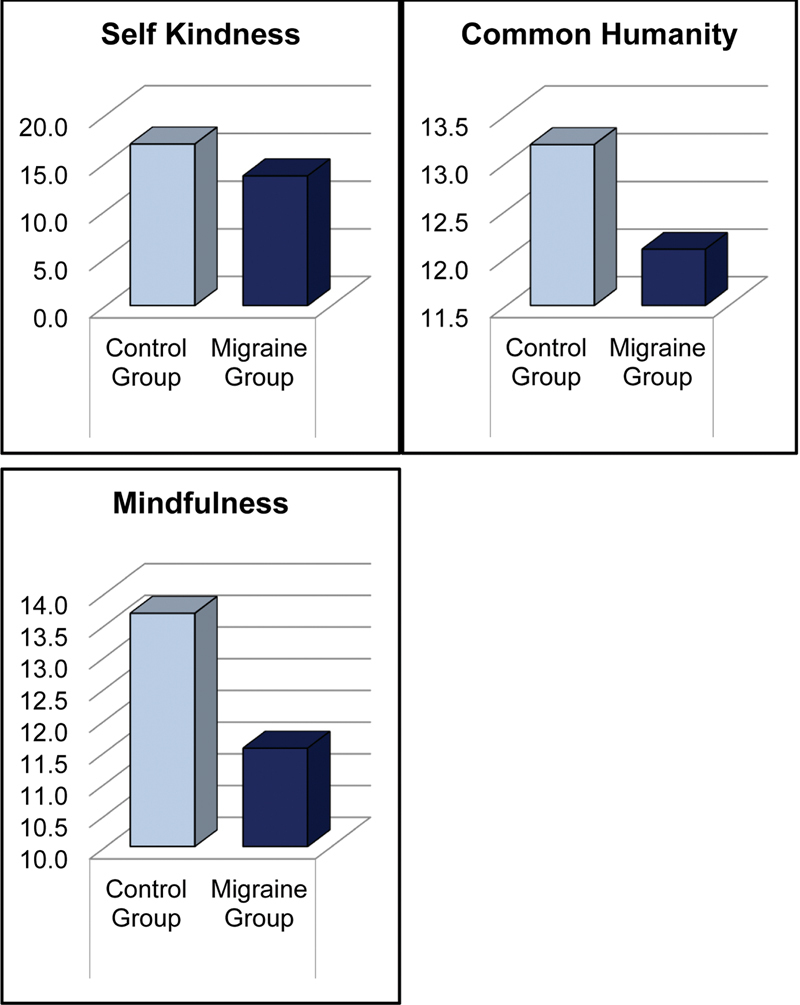
Self kindness, common humanity, and mindfulness scores in migraine and healthy controls.

**Figure 3:**
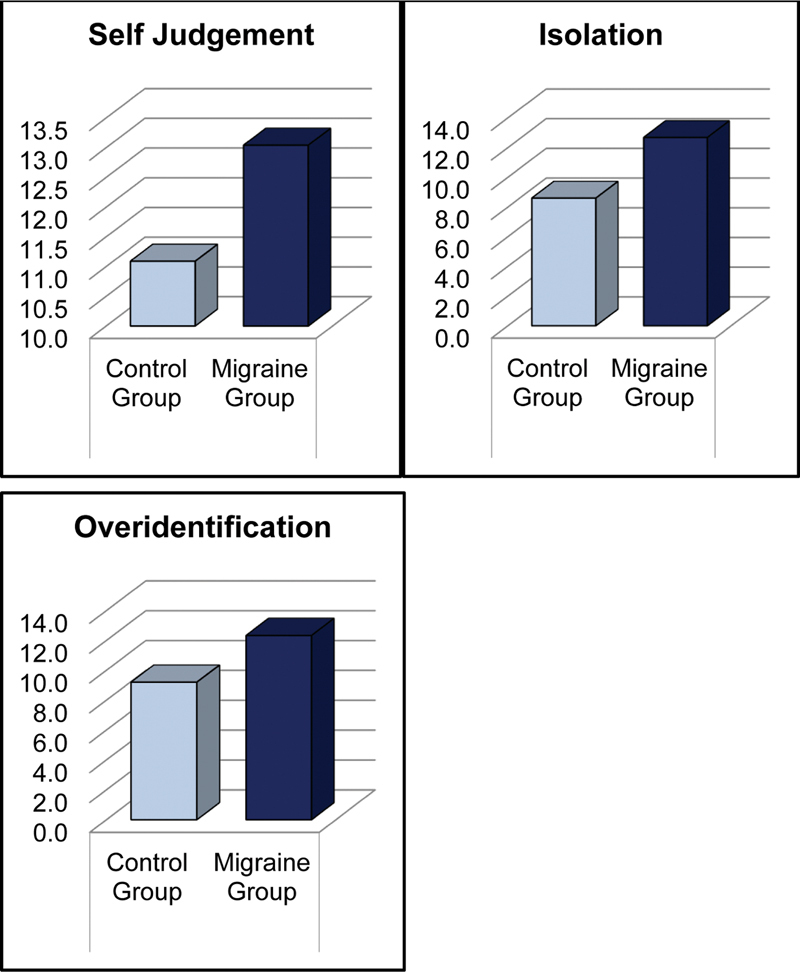
Self judgement, isolation, and overidentification scores in migraine and healthy controls.

There were no significant differences between groups regarding age, sex distribution, and education years (age: *p* = 0.421; sex: *p* = 0.316; education: *p* = 0.578). However, migraine patients had significantly higher depression and anxiety scores compared to healthy controls (BDI: *p* = 0.003; BAI: *p* = 0.018), as presented in Table 2 and Figure 1 . They also showed significantly lower scores in the self-kindness ( *p* < 0.001), common humanity ( *p* = 0.005), and mindfulness ( *p* < 0.001) subscales of the Self-Compassion Scale ( Table 2 , Figure 2 ) and significantly higher scores in self-judgment ( *p* = 0.001), isolation ( *p* < 0.001), and over-identification ( *p* < 0.001) subscales ( Table 2 , Figure 3 ).

**Table 2:** Baseline characteristics of study participants

		Healthy controls		Migraine group		*p* -value
		Mean ± SD / n (%)	Median	Mean ± SD / n (%)	Median	
Age		35.7 ± 9.8	35.5	36.7 ± 11.3	37.0	0.504 ^t^
Sex	Female	75 (83.3%)		84 (85.7%)		0.652 ^X2^
	Male	15 (16.7%)		14 (14.3%)		
Years of schooling		12.9 ± 3.6	12.5	12.8 ± 3.1	13.0	0.558 ^m^
BAI		8.7 ± 7.0	7.0	11.8 ± 8.7	9.0	**0.018** ^m^
BDI		6.7 ± 5.2	5.0	8.7 ± 5.5	7.5	**0.003** ^m^
SCS - self kindness		17.0 ± 4.1	16.5	13.6 ± 3.2	14.0	**<0.001** ^m^
SCS - self judgement		11.1 ± 3.7	10.5	13.0 ± 3.7	13.0	**0.001** ^m^
SCS - common humanity		13.2 ± 3.1	14.0	12.1 ± 2.8	12.0	**0.005** ^m^
SCS - isolation		8.6 ± 2.9	8.0	12.7 ± 3.5	13.0	**<0.001** ^m^
SCS - mindfulness		13.7 ± 3.1	13.0	11.6 ± 2.3	12.0	**<0.001** ^m^
SCS - overidentification		9.2 ± 2.7	8.0	12.3 ± 3.3	12.0	**<0.001** ^m^

Abbreviations: BAI, Beck anxiety inventory; BDI, Beck depression inventory; SCS, self-compassion scale; SD, standard deviation.

Notes: ^t^ Independent Samples t test; ^m^ Mann-Whitney U test; ^X2^ Chi-square test.

### Medication overuse in migraine patients

No significant differences were observed between migraine patients with and without medication overuse in terms of anxiety ( *p* = 0.711) and depression ( *p* = 0.087) scores. However, the medication overuse group had significantly lower scores in self-kindness ( *p* = 0.007), common humanity ( *p* = 0.046), and mindfulness ( *p* = 0.002) compared to those without ( Table 3A ).

**Table 3:** (A) Comparison of anxiety, depression and SC scores according to medication overuse status. (B) Comparison of anxiety, depression and SC scores according to presence of aura. (C) Comparison of anxiety, depression and SC scores according to migraine type (episodic vs. chronic)

A	Medication overuse (-) (n = 88)		Medication overuse (+) (n = 10)		*p* -value
	Mean ± SD	Median	Mean ± SD	Median	
BAI	11.6 ± 8.6	9.0	13.2 ± 10.3	12.5	0.711*
BDI	8.5 ± 5.6	7.0	10.4 ± 3.8	9.5	0.087*
SCS - self kindness	13.9 ± 3.2	14.0	11.1 ± 2.2	11.0	0.007*
SCS - self Judgement	12.9 ± 3.8	12.0	14.1 ± 2.2	14.0	0.174*
SCS - common humanity	12.3 ± 2.8	12.0	10.4 ± 2.2	11.0	0.046*
SCS - isolation	12.5 ± 3.5	13.0	14.2 ± 3.7	16.0	0.120*
SCS - mindfulness	11.8 ± 2.2	12.0	9.5 ± 1.8	9.5	0.002*
SCS - overidentification	12.2 ± 3.3	12.0	13.6 ± 3.4	14.5	0.124*
**B**	**Aura (-)** **(n = 77)**		**Aura (+)** **(n = 21)**		***p*** **-value**
	Mean ± SD	Median	Mean ± SD	Median	
BAI	11.7 ± 8.2	9.0	11.9 ± 10.6	8.0	0.664*
BDI	8.9 ± 4.6	9.0	8.0 ± 8.1	6.0	0.057*
SCS - self kindness	13.4 ± 3.1	14.0	14.4 ± 3.4	15.0	0.166*
SCS - self judgement	13.1 ± 3.4	13.0	12.9 ± 4.8	13.0	0.546*
SCS - common humanity	12.0 ± 2.8	12.0	12.3 ± 2.7	12.0	0.847*
SCS - isolation	13.0 ± 3.5	14.0	11.4 ± 3.1	11.0	0.022*
SCS - mindfulness	11.6 ± 2.2	12.0	11.5 ± 2.6	12.0	0.937*
SCS - overidentification	12.6 ± 3.3	13.0	11.3 ± 3.2	10.0	0.123 ^+^
**C**	**Episodic migraine** **(n = 89)**		**Chronic migraine** **(n = 9)**		***p*** **-value**
	Mean ± SD	Median	Mean ± SD	Median	
Anxiety score	11.5 ± 8.6	9.0	14.0 ± 10.6	14.0	0.534*
Depression score	8.6 ± 5.7	7.0	9.8 ± 3.5	8.0	0.202*
SCS - self kindness	13.9 ± 3.2	14.0	11.2 ± 2.3	11.0	0.016*
SCS - self judgement	12.9 ± 3.8	12.0	14.1 ± 2.4	14.0	0.203*
SCS - common humanity	12.3 ± 2.8	12.0	10.2 ± 2.2	11.0	0.033*
SCS - isolation	12.6 ± 3.5	13.0	13.8 ± 3.6	16.0	0.269*
SCS - mindfulness	11.8 ± 2.3	12.0	9.6 ± 1.9	10.0	0.006*
SCS - overidentification	12.2 ± 3.3	12.0	13.3 ± 3.5	14.0	0.230*

Abbreviations: BAI, Beck anxiety inventory; BDI, Beck depression inventory; SCS, Self-compassion scale; SD, standard deviation.

Notes: ^*^ Mann-Whitney U test; ^+^ Independent samples t test; significance threshold set at *p* < 0.05 (Bonferroni-adjusted).

### Aura presence

There were no significant differences in in anxiety ( *p* = 0.664) and depression ( *p* = 0.057) scores between patients with and without aura. Patients with aura showed significantly higher isolation scores ( *p* = 0.022), whereas no other SCS subscales differed significantly between groups ( Table 3B ).

### Episodic and chronic migraine groups

No significant differences were found between episodic and chronic migraine groups regarding anxiety ( *p* = 0.534), depression ( *p* = 0.202), isolation ( *p* = 0.269), or over-identification ( *p* = 0.230) scores. The chronic migraine group, however, exhibited significantly lower self-kindness ( *p* = 0.016), common humanity ( *p* = 0.033), and mindfulness ( *p* = 0.006) scores compared to the episodic migraine group ( Table 3C ).

### Correlation analysis

Age showed a significant positive correlation with depression (r = 0.435; *p* < 0.001) and isolation (r = 0.222; *p* = 0.028) scores, and a significant negative correlation with self-kindness (r = −0.260; *p* = 0.010) and common humanity (r = −0.205; *p* = 0.043).

Education level was negatively correlated with depression scores (r = −0.229; *p* = 0.023) and positively correlated with self-kindness (r = 0.207; *p* = 0.041) and common humanity (r = 0.219; *p* = 0.030). Migraine duration correlated positively with depression (r = 0.322; *p* = 0.001), self-judgment (r = 0.207; *p* = 0.041), isolation (r = 0.266; *p* = 0.008), and over-identification (r = 0.246; *p* = 0.014), and negatively with self-kindness (r = −0.235; *p* = 0.020) and common humanity (r = −0.200; *p* = 0.048). Finally, VAS pain scores correlated positively with anxiety scores (r = 0.239; *p* = 0.018), as shown in Table 4 .

**Table 4:** (A) Correlation analysis between anxiety, depression, self-kindness, and self-judgment and age, education, migraine duration, and pain severity. (B) Correlation analysis between common humanity, isolation, mindfulness, and overidentification and age, education, migraine duration, and pain severity

**A**		**BAI**	**BDI**	**SCS - self kindness**	**SCS - self judgement**
Age	r	0.148	0.435	−0.260	0.173 ^&^
	*p* -value	0.146	**<0.001**	**0.010**	0.089 ^&^
Education Years	r	−0.127	−0.229	0.207 ^*^	−0.084 ^&^
	*p* -value	0.212	**0.023**	**0.041**	0.411 ^&^
Migraine duration	r	0.094	0.322	−0.235	0.207 ^&^
	*p* -value	0.358	**0.001**	**0.020**	** 0.041 ^&^**
VAS score	r	0.239	−0.063	−0.069	0.077 ^&^
	*p* -value	**0.018**	0.536	0.501	0.454 ^&^
**B**		**SCS - common humanity**	**SCS - isolation**	**SCS - mindfulness**	**SCS - overidentification**
Age	r	−0.205	0.222	−0.102	0.172 ^&^
	*p* -value	**0.043**	**0.028**	0.315	0.090 ^&^
Education, years	r	0.219	−0.142	0.139	−0.176 ^&^
	*p* -value	**0.030**	0.165	0.173	0.083 ^&^
Migraine duration	r	−0.200	0.266	−0.139	0.246 ^&^
	*p* -value	**0.048**	**0.008**	0.172	** 0.014 ^&^**
VAS score	r	−0.090	0.034	−0.144	−0.012 ^&^
	*p* -value	0.376	0.736	0.159	0.909 ^&^

Abbreviations: BAI, Beck anxiety inventory; BDI, Beck depression inventory; SCS, self-compassion scale; VAS, visual analog scale.

Note: ^&^ Spearman' Correlation.

To examine whether migraine status was independently associated with SC beyond the effects of demographic characteristics and psychological symptoms, a series of hierarchical multiple regression analyses was conducted with SC as the dependent variable ( Table 5 ). Descriptive statistics for all variables are presented in Table 6 . A strong and negative correlation was found between SC and BAI scores (r = −0.45; *p* < 0.001), BDI scores (r = −0.48; *p* < 0.001), and migraine status (r = −0.48; *p* < 0.001). No significant correlations were observed between SC and age, gender, or years of education ( *p* > 0.05).

**Table 5:** Hierarchical linear regression predicting SC

Predictor		B	SE	β	t	*p-* value	95% CI for B
Step 1	Age	−0.11	0.12	−0.07	−0.88	0.378	−0.35, 0.13
	Gender (1 = female, 2 = male)	4.40	3.30	0.10	1.33	0.184	−2.12, 10.92
	Years of education	0.24	0.39	0.05	0.61	0.543	−0.53, 1.00
Step 2	BAI	−0.66	0.13	−0.33	−5.09	< 0.001	−0.92, −0.41
	BDI	−1.15	0.20	−0.38	−5.90	< 0.001	−1.53, −0.76
Step 3	Migraine status (vs. control)	−12.18	1.81	−0.38	−6.74	< 0.001	−15.75, −8.62

Abbreviations: BAI, Beck anxiety inventory; BDI, Beck depression inventory; SE, standard error.

Note: Dependent variable = total self-compassion score (sum of 26 items). Group was dummy coded (0 = control, 1 = migraine).

**Table 6:** Descriptive statistics and Pearson correlations among study variables (N = 188)

Variable	Mean	SD	1	2	3	4	5	6	7
1. SC	84.74	16.26	—						
2. Age	36.21	10.60	−0.08	—					
3. Gender ^a^	1.15	0.36	0.10	0.08	—				
4. Education (years)	12.85	3.30	0.08	−0.36***	0.07	—			
5. BAI	10.31	8.09	−0.45***	0.09	−0.19**	−0.06	—		
6. BDI	7.74	5.44	−0.48***	0.18**	−0.13*	−0.04	0.33***	—	
7. Group ^b^	0.52	0.50	−0.48***	0.05	−0.03	−0.01	0.19**	0.18**	—

Abbreviations: BAI, Beck anxiety inventory; BDI, Beck depression inventory; SC, self-compassion; SD, standard deviation.

Note: Values are represented as Pearson correlation coefficients; ^a^ gender was coded as 1 = female, 2 = male; ^b^ group was dummy-coded as 0 = control, 1 = migraine; * *p* < 0.05; ** *p* < 0.01; *** *p* < 0.001.

In Step 1 of the hierarchical multiple regression analyses, age, gender, and years of education did not significantly predict SC, ( *R ^2^* = 0.019; adjusted *R ^2^* = 0.003; *F* [3, 184] = 1.17; *p* = 0.323). None of the demographic variables were significant independent predictors ( *p* > 0.05). In Step 2, the addition of BAI and BDI scores resulted in a substantial and statistically significant increase in explained variance, (Δ *R ^2^* = 0.315, Δ *F* [2, 182] = 43.11; *p* < 0.001). Higher BAI ( *B* = −0.66; 95% CI: −0.92, −0.41; *p* < 0.001) and higher BDI scores ( *B* = −1.15; 95% CI: −1.53, −0.76; *p* < 0.001) were both independently associated with lower SC scores. In Step 3, migraine status was entered into the model and explained additional variance in SC beyond demographics and psychological symptoms (Δ *R ^2^* = 0.134; Δ *F* [1, 181] = 45.48; *p* < 0.001). Migraine status emerged as a strong independent predictor of SC ( *B* = −12.18; 95% CI: −15.75, −8.62; *p* < 0.001), indicating substantially lower SC scores among individuals with migraine compared to healthy controls.

In the final model, BAI ( *B* = −0.55; 95% CI: −0.79, −0.32; *p* < 0.001), BDI scores ( *B* = −1.00; 95% CI: −1.35, −0.66; *p* < 0.001), and migraine status remained significant predictors, whereas age, gender, and years of education were not ( *p* > 0.05). The final model explained 46.8% of the variance in SC ( *R ^2^* = 0.468; adjusted *R ^2^* = 0.45).

## DISCUSSION

The present study aimed to compare SC levels between individuals with migraine and healthy controls, as well as across clinically relevant migraine subgroups, including patients with and without medication overuse, migraine with and without aura, and episodic versus chronic migraine. To our knowledge, few studies have examined the subdomains of SC in migraine populations.

Our main finding is that migraine patients exhibited lower overall SC compared to healthy controls. Specifically, they scored higher on the negative subdomains of self-judgment, isolation, and over-identification, and lower on the positive subdomains of self-kindness, common humanity, and mindfulness. Despite excluding participants with clinically diagnosed depression or anxiety, migraine patients showed higher subclinical levels than controls, consistent with previous reports of increased psychiatric comorbidity in this population. 26 Given the inverse association between SC and mood symptoms, it remains unclear whether deficits in SC are intrinsic to migraine or partly secondary to subclinical emotional distress. 27

Within the migraine cohort, patients without medication overuse scored higher in self-kindness, common humanity, and mindfulness. Despite awareness of the risks, this issue remains prevalent and may reflect habitual or compulsive use. 28 Our findings suggest that higher SC and mindfulness may be associated with more adaptive coping strategies and reduced reliance on excessive analgesic use.

Patients with aura exhibited higher isolation scores than those without aura. This may reflect the unpredictable nature of aura and associated neurological symptoms, which can increase vulnerability and perceived social disconnection. Behavioral strategies aimed at preventing attacks, such as avoiding crowds or emotional triggers, may further reinforce feelings of isolation. 29

When comparing episodic and chronic migraine, there were no significant differences in anxiety or depression; however, chronic migraine patients demonstrated lower self-kindness, common humanity, and mindfulness. This may suggest that deficiencies in SC can be more closely linked to headache frequency or chronicity than to mood symptoms. Chronic pain has well-documented negative effects on quality of life, and emerging evidence indicates that SC-based interventions can improve coping and psychological outcomes in these populations. 30 31

The bidirectional relationship between emotional regulation and migraine is increasingly recognized. 32 Higher SC may facilitate adaptive emotional regulation through its soothing and affiliative qualities, potentially mitigating stress-related physiological responses such as interleukin-6 and cortisol elevations. 17 33 34 In this context, our findings indicate that lower scores are associated with greater migraine chronicity and emotional distress. However, given the cross-sectional design, these results should be interpreted as associative rather than explanatory, and no causal or mechanistic inferences can be drawn. Longitudinal and experimental studies are needed to clarify the temporal and biological pathways linking SC, emotional processes, and migraine. 35

Importantly, after regression analyses, migraine status remained independently associated with lower SC even after controlling for demographic variables, depression, and anxiety. Although depression and anxiety symptoms were, as expected, strong independent predictors of lower SC, migraine itself accounted for a substantial proportion of additional variance. This finding suggests that reduced SC in migraine cannot be explained solely as a secondary consequence of emotional distress, but may instead reflect a distinct psychological vulnerability or resilience-related factor associated with the migraine condition.

In recent years, mindfulness-based interventions have been increasingly used in migraine management, and evidence has emerged showing beneficial effects on headache-related disability, emotional distress, and quality of life. 11 12 While mindfulness represents a fundamental component of SC, current findings suggest that migraine patients may experience impairments not only in mindfulness but also in SC, shared sense of humanity, and increased self-criticism and isolation. This broader pattern suggests that interventions focusing solely on mindfulness may not fully address the self-relational difficulties observed in this population. Therefore, compassion-focused and SC-enhancing approaches may provide additional benefits by directly targeting self-criticism, isolation, and over-identification, which are prominent characteristics in our migraine group. 31 32

This study has several limitations. First, recruitment from a university headache clinic may have resulted in a sample with more severe or treatment-resistant migraines, limiting generalizability. Second, the predominantly female sample restricts analyses of potential sex differences in SC. Third, the cross-sectional design precludes causal inference. Finally, reliance on self-report questionnaires introduces potential recall and reporting biases. While subgroup sizes were modest, this study serves as a pilot and provides a foundation for future research. Longitudinal studies with larger, more diverse samples are needed to validate these findings and evaluate the potential efficacy of compassion-focused interventions in reducing migraine frequency or severity.

In conclusion, this study demonstrates that migraine patients exhibit lower SC across all subdomains compared to healthy controls, with chronic migraine, aura presence, and medication overuse being associated with more pronounced deficits. These findings highlight SC as a relevant psychological correlate of migraine and support the further exploration of compassion-based strategies in the management of this condition.
